# Development and Exploratory Evaluation of a Habitual Caffeine Intake-Based Index to Characterize the Ergogenic Response to Caffeine in Wrestlers

**DOI:** 10.3390/nu18213625

**Published:** 2026-10-31

**Authors:** Mostafa Khodadoost, Raoof Negaresh, Reza Bagheri, Shihab Al-Din Ahmed Said Al-Reiamy, Motahare Mokhtarzade, Juan Del Coso

**Affiliations:** 1 Department of Physical Education, Arv.C., Islamic Azad University, Abadan, Iran; 2 Department of Physical Education, Farhangian University, Tehran, Iran; raoof.negaresh@cfu.ac.ir; 3 International UNESCO Center for Health-Related Basic Sciences and Human Nutrition, Mashhad University of Medical Sciences, Mashhad, Iran; 4 Research in Medical Research Center (MRC), Sultan Qaboos University, Muscat, Oman; 5 Department of Sport Sciences, Faculty of Humanities, Tarbiat Modares University, Tehran, Iran; 6 Sport Sciences Research Centre, Rey Juan Carlos University, Madrid, Spain

**Keywords:** caffeine, habitual caffeine intake, ergogenic response, adverse effects, personalized nutrition

## Abstract

**Objectives:** This secondary exploratory analysis of a randomized, double-blind, placebo-controlled crossover trial developed and compared indices linking administered caffeine dose to habitual intake to characterize variability in performance and side effects in wrestlers. **Methods:** Seventy-two male wrestlers completed a randomized, double-blind, placebo-controlled crossover trial in which they ingested caffeine (4 mg·kg^−1^) or placebo 60 min before vertical jump and wrestling-specific testing. Three indices were calculated: DAR, expressing the administered dose as a multiple of the habitual intake; DDI, quantifying the additional caffeine received above habitual intake; and DNI, expressing this additional intake as a proportion of the administered dose. Hierarchical regression models were used to examine whether each index provided incremental explanatory value beyond habitual caffeine intake and absolute caffeine dose. **Results:** In exploratory hierarchical models adjusted for habitual caffeine intake and absolute dose, DAR was associated with additional explained variance for the change in total throws (ΔR^2^ = 0.249, *p* < 0.001), the wrestling performance index (ΔR^2^ = 0.321, *p* < 0.001), the Caffeine Utility Index (ΔR^2^ = 0.160, *p* < 0.001), and side effect severity (ΔR^2^ = 0.072, *p* = 0.008), but not for the change in vertical jump performance (ΔR^2^ = 0.004, *p* = 0.586). DDI provided significant incremental explanatory value for the change in total throws (ΔR^2^ = 0.061, *p* = 0.027) and the wrestling performance index (ΔR^2^ = 0.067, *p* = 0.022). Exploratory quadratic analyses identified a theoretical DAR vertex range between 2.25 and 2.81. **Conclusions:** These findings support DAR as the principal index for prospective evaluation, but they do not establish a dosing prescription. DAR is not intended to replace body-mass-based dosing (mg·kg^−1^); rather, it provides a complementary reference dimension that should be applied within established safety limits.

## 1. Introduction

Caffeine is one of the most widely consumed ergogenic aids in sport, and substantial evidence has demonstrated that its ingestion can enhance endurance performance, anaerobic capacity, muscular power, speed, alertness, and cognitive function [1,2,3]. Consequently, caffeine is among the few nutritional supplements whose ergogenic efficacy has been consistently supported by a broad body of research conducted by experts in sports nutrition and exercise science [4]. It is therefore widely used across numerous sporting disciplines as a legal strategy to enhance athletic performance. In recent years, researchers have increasingly shifted their focus from generalized nutritional recommendations toward personalized sports nutrition. This approach is based on the premise that a single supplementation strategy is unlikely to be optimal for all athletes and that individual characteristics should be considered when developing nutritional interventions [5,6]. Nevertheless, despite this paradigm shift, current caffeine recommendations continue to rely primarily on relative body mass-based doses (typically 3–6 mg·kg^−1^), with relatively little consideration given to athletes’ habitual caffeine consumption [5,7].

Despite its well-established ergogenic effects, athletes exhibit considerable inter-individual variability in their responses to caffeine supplementation, even when the dose is prescribed relative to body mass. Such variability may reflect differences in caffeine pharmacokinetics and pharmacodynamics, including polymorphisms affecting caffeine metabolism and adenosine signaling, particularly CYP1A2 rs762551 and ADORA2A rs5751876 [8,9], as well as sex [10], training status [11,12], timing of ingestion [13,14], sport-specific demands [15], expectancy [16], sleep [14,17], and habitual caffeine exposure [12]. Importantly, a limited response in a single trial should not be interpreted as definitive evidence of caffeine non-responsiveness because day-to-day biological and measurement variability can also contribute to the observed response [18]. In other words, an athlete’s response to caffeine is likely shaped by the interaction between the person, the dose, and the competitive context rather than by body mass alone.

Habitual caffeine intake is especially relevant because repeated exposure may modify the physiological response to a subsequent dose. At physiologically relevant concentrations, caffeine acts primarily as a competitive antagonist of adenosine A_1_ and A_2_A receptors, reducing adenosine-mediated inhibition of neuronal activity. Repeated caffeine exposure can induce compensatory adaptations in the adenosinergic system, including altered receptor expression and downstream signaling, which provides a plausible mechanism for tolerance; however, much of the mechanistic evidence derives from experimental and animal models, and its contribution to exercise habituation in humans remains uncertain [19,20,21]. Human evidence also differs according to study design. Cross-sectional comparisons of low and high habitual consumers often report similar acute ergogenic effects, whereas longitudinal studies in which caffeine is consumed repeatedly over days or weeks suggest that the magnitude of the ergogenic response can attenuate with repeated exposure in at least some exercise contexts [5,22,23,24,25]. This discrepancy suggests that “habitual intake” is not a single biological construct: the amount habitually consumed, the duration and pattern of exposure, recent abstinence, and the acute dose relative to usual intake may all influence the observed response [12]. This is why habitual intake is better viewed as part of an exposure history than as a simple low-versus-high consumer label.

An important consideration is caffeine withdrawal. In habitual consumers, 24-h abstinence can produce withdrawal symptoms (headache, fatigue, reduced alertness) that impair placebo performance and may be partially reversed by subsequent caffeine administration [26,27]. Because habitual intake is the central exposure variable in this analysis, withdrawal effects could contribute directly to the observed association between habitual intake and the caffeine–placebo response, potentially reflecting withdrawal reversal rather than a purely ergogenic effect.

This issue may be particularly relevant in combat sports, in which performance depends on repeated explosive actions, high anaerobic power, technical execution under fatigue, perceptual control, and rapid recovery between intense efforts. Wrestling-specific research has shown that caffeine can affect performance during simulated competition and sport-specific testing, but most studies have prescribed caffeine as a fixed dose relative to body mass without explicitly integrating habitual caffeine exposure into the dosing framework [15,18,22,23,24]. Evidence from other intermittent and strength-power sports likewise indicates substantial variation in both ergogenic and adverse responses despite standardized mg·kg^−1^ dosing [28,29,30]. Thus, the unanswered question is not simply whether caffeine is ergogenic, but whether the meaning of a given experimental dose changes according to the athlete’s usual exposure. A metric that explicitly relates administered dose to habitual intake could provide a practical way to test this proposition. For wrestlers, that distinction is practically important because a small gain in repeated high-intensity work may be offset if the same exposure increases symptoms that interfere with technical control or recovery.

Accordingly, the current study aimed to develop and compare candidate indices that relate the administered caffeine dose to habitual caffeine intake, and to examine whether these indices provide incremental explanatory value beyond their constituent variables, habitual caffeine intake and absolute caffeine dose, for wrestling-specific performance and side effect burden. Three candidate formulations were evaluated: the Dose-to-Habitual Intake Ratio (DAR), Dose Difference Index (DDI), and Dose Novelty Index (DNI). The a priori practical objective was to identify the index that showed the strongest explanatory signal for future prospective evaluation rather than to propose three separate prescription tools.

## 2. Materials and Methods

### 2.1. Participants

Seventy-five male wrestlers volunteered to participate in this randomized, double-blind, placebo-controlled crossover study. As this was a secondary analysis of a parent trial, no a priori sample size calculation was performed. The sample size was determined by the parent trial. Participants were recruited from regional wrestling clubs and competitive teams through direct contact with coaches and local wrestling federations. Eligibility criteria included: (a) age between 18 and 40 years; (b) a minimum of three years of continuous wrestling training; (c) regular participation in organized training (≥3 sessions/week); and (d) current involvement in freestyle or Greco-Roman wrestling at the amateur, semi-professional, or professional level. Exclusion criteria included: (a) history of any diagnosed cardiovascular, metabolic, neurological, respiratory, or gastrointestinal disease, (b) injury limiting maximal exercise performance, (c) illness within the two weeks prior to the study, (d) smoking, (e) using medication that could interfere with study outcomes, and (f) rapid weight loss within the month preceding the study. The study protocol was approved by the Ethics Committee of Abadan University of Medical Sciences (ethical code: IR.ABADAN.REC.1404.131) and was conducted in accordance with the principles of the Declaration of Helsinki. All participants received detailed verbal and written explanations of the study procedures and provided written informed consent before participation.

The parent trial was not prospectively registered. We intended to register the trial in the Iranian Registry of Clinical Trials (IRCT) before enrollment; however, wartime armed conflict and severe internet restrictions prevented access to the online registration system. By the time access was restored, enrollment had commenced, and the IRCT does not permit retrospective registration. The trial was therefore retrospectively registered in ClinicalTrials.gov under registration number NCT07889661 on 16 October 2026.

### 2.2. Experimental Procedures

Following eligibility screening and familiarization, participants completed a randomized, double-blind, placebo-controlled crossover trial consisting of two experimental sessions separated by a 7-day washout period to minimize potential carryover effects. Before each experimental session, participants were instructed to avoid strenuous physical exercise for 48 h, alcohol consumption for 24 h, and caffeine-containing foods, beverages, supplements, and medications for 24 h. Withdrawal symptoms were not formally assessed before each experimental session. They were also asked to maintain their normal dietary habits throughout the study and to replicate their pre-test meal before each testing session. During one session, participants ingested 4 mg/kg of caffeine, whereas during the other session they received a placebo. The order of treatments was determined using a computer-generated randomization sequence and was concealed from both participants and investigators until completion of all data analyses.

All experimental sessions were conducted at the same time of day for each participant to minimize potential circadian influences. Upon arrival at the laboratory, participants confirmed compliance with all pre-test instructions and completed a brief health screening questionnaire. Body mass was recorded before supplementation to calculate the individualized caffeine dose. Participants then consumed the assigned capsules with approximately 250 mL of water under researcher supervision. Athletes rested for 60 min and then completed a standardized warm-up consisting of 10 min of light jogging, dynamic stretching, and wrestling-specific movement drills identical to those routinely performed before training sessions. After the warm-up, participants completed the performance testing battery in a standardized order. Vertical jump (VJ) performance was assessed first to minimize fatigue-related interference. Athletes then performed the wrestling-specific performance test. Following completion of performance testing, participants completed a validated questionnaire assessing the presence and severity of caffeine-related side effects. The current study represents a secondary analysis of data collected within this trial. Data from the same parent study have also been used in a companion manuscript examining differences in caffeine responses across wrestling weight classes and the resulting absolute caffeine exposure. The present analysis addresses a distinct objective by developing and comparing indices that relate the administered caffeine dose to habitual caffeine intake.

### 2.3. Assessment of Habitual Caffeine Intake

Habitual caffeine intake was assessed using the caffeine intake assessment tool originally developed by Bühler et al. [31], which has been previously used in athletic populations [32,33]. Participants reported their usual consumption of caffeine-containing products (different types of coffee, tea, energy drinks, soft drinks, chocolate, and caffeine-containing supplements) over the previous month, including daily, weekly, and monthly consumption patterns. Standard household measures were used to estimate portion sizes, and exact brands of products were identified where possible. A qualified nutritionist estimated the daily caffeine intake (mg/day) for each participant using standard nutritional databases, covering the four weeks preceding the experimental trials. The original FFQ was adapted for the present study to include caffeine-containing products commonly consumed in Iran (e.g., Turkish coffee, black tea) and to capture the typical consumption patterns of Iranian male wrestlers. To maximize the variability in habitual caffeine exposure, no minimum habitual caffeine intake was required for study participation.

### 2.4. Caffeine Supplementation

The present study represents a secondary analysis of data collected within this trial. Data from the same parent study have been used in a companion manuscript examining differences in caffeine responses across wrestling weight classes. The current analysis addresses a distinct objective by developing and comparing indices that relate the administered caffeine dose to habitual caffeine intake. Briefly, athletes completed two experimental trials, ingested anhydrous caffeine at a dose of 4 mg·kg^−1^ body mass or received an identical placebo capsule containing dextrose. Both caffeine and placebo were encapsulated in opaque gelatin capsules identical in appearance, size, weight, and color to ensure effective blinding. The caffeine dose was individually calculated according to each athlete’s body mass measured on the testing day. Capsules were administered with approximately 250 mL of water 60 min before performance testing. The absolute dose administered ranged from 228 to 556 mg, depending on individual body weight. Blinding effectiveness was assessed immediately after each experimental session by asking participants to indicate whether they believed they had received caffeine or placebo (Supplementary File Table S1). Treatment allocation codes were concealed until completion of data collection and statistical analyses.

### 2.5. Measurements

#### 2.5.1. Athlete Characteristics

Participants completed a standardized questionnaire to provide demographic and sport-related information, including age, wrestling style (freestyle or Greco-Roman), training history, and average weekly training volume. Body mass was measured immediately before each experimental session using a calibrated digital scale (Seca GmbH, Hamburg, Germany), with participants wearing wrestling clothing and no shoes. Standing height was measured at the screening session using a wall-mounted stadiometer (Seca GmbH, Hamburg, Germany).

Competitive level was classified into three categories according to recent competitive experience and performance history. Professional wrestlers were defined as athletes who had represented the Iranian national team in international competitions or had been official members of the national team within the previous three years or who, during the preceding competitive season, competed in the national premier wrestling league. Semi-professional wrestlers were those who had not represented the national team during the previous three years and had not served as regular athletes in the national premier wrestling league during the preceding season or were actively competing in other national-level leagues or competitions. Amateur wrestlers were defined as athletes who had competed exclusively in provincial-level competitions during the previous three years or had not participated in any official wrestling competitions during that period. Training history was defined as the number of years of wrestling practice, while weekly training volume represented the average number of structured wrestling training hours performed during the previous three months.

#### 2.5.2. Athletic Performance

Athletic performance was evaluated using two complementary tests assessing lower-body explosive power and wrestling-specific performance. All testing was performed on a standard wrestling mat under identical environmental conditions and at the same time of day for both experimental sessions.

**Vertical Jump Test:** Lower-body explosive power was assessed using the Sargent VJ test (wall-mounted reach method). Before testing, participants stood barefoot alongside a wall and reached upward with the dominant arm fully extended while keeping both feet flat on the floor. The highest fingertip position was marked as the standing reach height. Participants then performed a maximal countermovement jump using a self-selected countermovement depth and touched the wall at the highest possible point [34,35]. Jump height (cm) was calculated as the difference between the standing reach and maximal jump reach. Three maximal attempts were performed with 60 s of passive recovery between attempts, and the highest value was retained for statistical analyses. Standardized verbal encouragement was provided during each trial.

**Wrestling-Specific Fitness Test (SWFT):** Wrestling-specific performance was assessed using the SWFT, a validated field test designed to reproduce the intermittent high-intensity physiological demands of wrestling competition [36,37,38]. The SWFT consisted of three 30-s maximal work bouts separated by 20-s passive recovery intervals. During each work period, participants performed as many technically valid throws as possible using a wrestling dummy (Suples Ltd., Boise, ID, USA). Dummy mass was selected according to the wrestler’s official body-mass category: 22 kg for athletes weighing < 75 kg, 27 kg for athletes weighing 75–90 kg, and 32 kg for athletes weighing > 90 kg [36,37,38]. Freestyle wrestlers performed a modified arm throw (fireman’s carry), whereas Greco-Roman wrestlers performed the suplex technique. Successful throws were independently counted by three experienced wrestling referees/coaches holding official national certification. A throw was considered valid only when it satisfied the technical scoring criteria defined by United World Wrestling (UWW) regulations. Any disagreement between assessors was resolved immediately by consensus.

The total number of successful throws (Tn) completed across the three work intervals was considered the primary performance outcome. Heart rate was recorded immediately after the test (HR_0_) and after 1 min of passive recovery (HR_1_) using a Polar heart-rate monitor (Polar Electro, Kempele, Finland). Immediately following the test, participants rated their perceived exertion using the 6–20 Borg Rating of Perceived Exertion (RPE) scale. To minimize the influence of body-mass differences across weight categories, the total number of throws was additionally normalized to body mass:

$$\mathrm{TnW}=\frac{\mathrm{Tn}}{\mathrm{Body mass}(\mathrm{kg})}$$

Additionally, a wrestling-specific Performance Index was calculated as:

$$\mathrm{Performance Index}=\frac{HR_{0}+HR_{1}}{\mathrm{Tn}}$$

where lower values indicate superior wrestling-specific performance [36,37,38]. One week before the first experimental session, all participants completed a familiarization session during which they practiced both performance tests and became accustomed to the testing procedures, heart-rate monitoring equipment, and RPE scale. Each athlete completed at least two practice trials of the SWFT to minimize learning effects during the experimental sessions.

#### 2.5.3. Side Effects

Side effects were assessed using the Questionnaire for the Assessment of Side Effects, which has been previously used to evaluate self-reported adverse responses following caffeine ingestion in athletic populations [39,40,41]. Participants completed the questionnaire immediately after the completion of the final performance test, thereby capturing all side effects experienced from the time of capsule ingestion until the end of the experimental session.

The questionnaire evaluated the occurrence of the following symptoms: vigor/activeness, anxiety, headache, dizziness, nervousness, irritability, tachycardia, abdominal discomfort, increased urine output, muscle soreness, and fatigue. For each symptom, participants first indicated whether it was experienced (yes/no). If present, symptom severity was subsequently rated using a 5-point Likert scale ranging from 1 (mild) to 4 (very severe). The number of reported side effects was calculated as the total number of symptoms experienced during the experimental session, whereas the side effect severity score was calculated as the sum of the severity ratings for all reported symptoms, providing an overall measure of the magnitude of adverse responses following supplementation. Importantly, vigor/activeness was not included in either the side effect frequency or the side effect severity score, because it represents a potentially desirable effect of caffeine rather than an adverse effect in wrestling.

### 2.6. Development of Personalized Caffeine Indices

**Figure 1:**
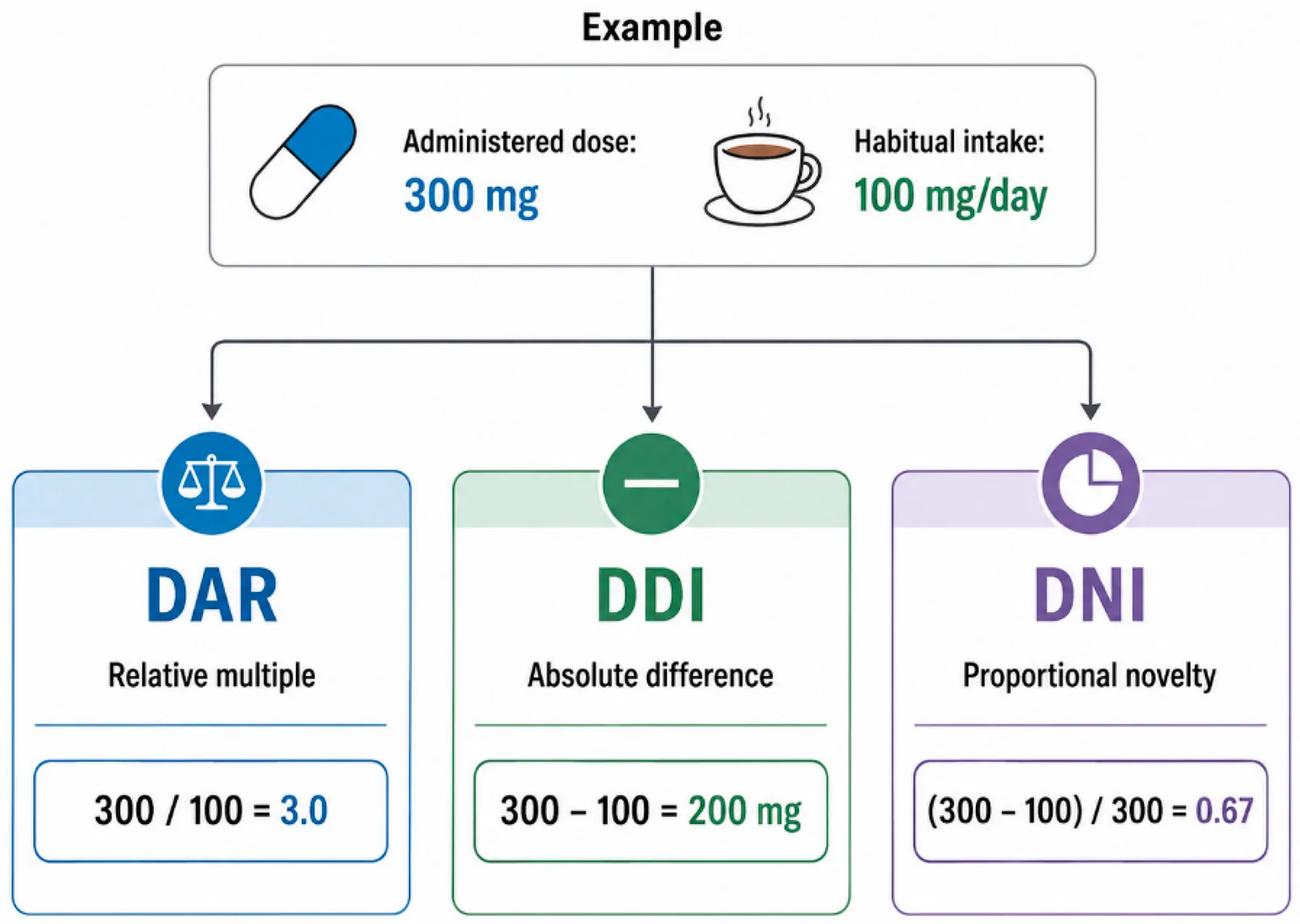
Calculation of the three personalized caffeine indices. Dose-to-Habitual Intake Ratio (DAR; administered dose/habitual intake), Dose Difference Index (DDI; administered dose–habitual intake), and Dose Novelty Index (DNI; [administered dose–habitual intake]/administered dose). The example assumes a 300 mg caffeine dose and a habitual intake of 100 mg/day.

To examine whether the relationship between habitual caffeine exposure and the experimental dose could be represented by a practical individualized metric, three candidate indices were calculated and compared (Figure 1). The purpose of comparing three formulations was to identify the most informative and interpretable candidate for subsequent development, rather than to propose three independent prescription systems.

(1) The DAR was calculated as the ratio of the caffeine dose to the athlete’s habitual daily caffeine intake:

$$\mathrm{DAR}=\frac{\mathrm{Caffeine dose}(\mathrm{mg})}{\mathrm{Habitual caffeine intake}(\mathrm{mg}/\mathrm{day})}$$

This ratio represents the relative magnitude of the administered dose compared with the athlete’s habitual caffeine exposure. Habitual caffeine intake was expressed as the average daily intake (mg/day) and was assessed using the FFQ (Section 2.3). Habitual caffeine intake was operationalized as the estimated amount of caffeine (mg) typically consumed over a representative 24-h day. Accordingly, DAR expresses the acute dose as a multiple of this 24-h reference intake. The ratio should therefore be interpreted as the number of days’ worth of habitual intake represented by the acute dose, rather than as a ratio of dimensionally identical units. Higher DAR values indicate that the experimental dose represents a larger increase over the athlete’s normal caffeine consumption.

(2) The DDI was calculated as:

DDI = Caffeine dose (mg) − Habitual caffeine intake (mg/day)

This index reflects the absolute difference between the administered dose (mg) and the estimated amount typically consumed over a representative 24-h day (mg). Expressing habitual intake as a 24-h amount allows DDI to be interpreted in milligrams, although the ‘per day’ element denotes the reference period rather than a separate unit. Because the experimental dose itself was calculated from body mass, DDI should not be interpreted as mathematically independent of body mass.

(3) The DNI was calculated as:

$$\mathrm{DNI}=\frac{\mathrm{Caffeine dose}(\mathrm{mg})-\mathrm{Habitual caffeine intake}(\mathrm{mg}/\mathrm{day})}{\mathrm{Caffeine dose}(\mathrm{mg})}$$

The DNI represents the fraction of the administered dose that exceeds habitual intake. Values approaching zero indicate that the administered dose is similar to habitual intake, whereas values approaching one indicate a markedly greater exposure than usual. Because DNI is algebraically related to DAR (DNI = 1 − 1/DAR), it was interpreted as an alternative scale transformation of the same dose–habitual intake relationship rather than as a conceptually independent candidate.

All three indices were calculated individually for each athlete using the caffeine dose administered during the experimental session and the estimated habitual caffeine intake. Because DAR is a ratio-based index, very low habitual caffeine intake values can mathematically amplify the ratio and produce extreme DAR values. To avoid undefined or disproportionately large DAR values, a floor value of 30 mg/day was applied to habitual caffeine intake for the calculation of DAR. This threshold was chosen a priori as a pragmatic approach to preserve the relative interpretation of the index while preventing mathematical amplification at very low intake values.

### 2.7. Caffeine Utility Index

To provide an integrated measure of the overall utility of caffeine supplementation, a Caffeine Utility Index (CUI) was developed. Because the study included performance variables measured on different scales, all variables were first standardized using z-scores to place them on a common metric with a mean of zero and a standard deviation of one.

The CUI was constructed by combining the standardized ergogenic outcomes while accounting for adverse effects according to the following equation:

CUI = (ZΔTn + ZΔVJ) − ZΔSES

where ZΔTn is the standardized change in throwing number, ZΔVJ is the standardized change in vertical jump performance, and ZΔSES is the standardized change in side effect severity score.

Because greater improvements in throwing performance and VJ represent desirable outcomes, their standardized scores were added, whereas the standardized side effect severity score was subtracted because greater adverse effects reduce the practical utility of caffeine supplementation. Accordingly, higher CUI values indicate a more favorable balance between ergogenic benefits and side effects, whereas lower or negative values indicate that the overall utility of caffeine supplementation is reduced by limited performance enhancement and/or greater side effect burden.

### 2.8. Statistical Analysis

Statistical analyses were performed using IBM SPSS Statistics version 27, and statistical significance was established at *p* < 0.05. Primary and secondary outcomes. The prespecified primary outcomes were ΔTn, ΔVJ, and CUI. Secondary outcomes included the wrestling performance index, side effect frequency, side effect severity, heart rate and RPE. To compare performance outcomes between the caffeine and placebo conditions, paired-samples *t*-tests were performed for normally distributed variables, whereas the Wilcoxon signed-rank test was used for variables that violated normality assumptions. Effect sizes were calculated as Cohen’s d for paired *t*-tests and as the effect size coefficient (r) for Wilcoxon tests. Pearson product-moment correlation coefficients were calculated for normally distributed variables to examine the associations between the personalized caffeine indices (DAR, DDI, and DNI) and performance outcomes. For side effect frequency and side effect severity, which were not normally distributed or were ordinal, Spearman’s rank correlation coefficients were used.

To determine whether the personalized caffeine indices provided incremental explanatory value beyond their constituent variables, hierarchical multiple linear regression analyses were conducted using a three-model structure. For each outcome, Model 1 (baseline) included age, training history, competitive level, and wrestling style. Model 2 additionally included habitual caffeine intake and absolute caffeine dose. Model 3 further added DAR, DDI, or DNI (each entered in a separate parallel model). Competitive level was dummy-coded with amateur as the reference category (semi-professional vs. amateur; professional vs. amateur), and wrestling style was coded as Greco-Roman vs. freestyle. Both variables were treated as categorical. For all regression models, we examined residual normality, homoscedasticity, multicollinearity (variance inflation factor, VIF), and influential observations.

As a supplementary analysis, we also examined whether the personalized caffeine indices provided additional explanatory value beyond conventional participant characteristics. In each model, participant characteristics were entered in the first block. Caffeine dose, habitual caffeine intake, DAR, DDI, and DNI were subsequently entered individually in separate models. Full results of this supplementary analysis are provided in the Supplementary File (Table S2). To evaluate the robustness of the quadratic regression models, bootstrap resampling with 5000 samples and bias-corrected and accelerated (BCa) 95% confidence intervals was performed for the regression coefficients of the quadratic models. Bootstrap estimates were used to assess the stability of the nonlinear associations while accounting for sampling variability.

To examine whether the relationship between DAR and ergogenic response was linear or curvilinear, quadratic regression models were fitted using centered DAR values and their squared term. Centering was performed to reduce multicollinearity between the linear and quadratic predictors. The vertex of the quadratic function was estimated according to:

$$\mathrm{Explatory Vertex of DAR}={\mathrm{Mean}}_{\mathrm{DAR}}-\frac{b_{1}}{2b_{2}}$$

where b_1_ and b_2_ represent the unstandardized regression coefficients of the centered linear and quadratic terms, respectively.

To facilitate practical interpretation of the identified exploratory vertex of the DAR range, athletes were additionally categorized into quartiles based on the DAR values. Differences among quartiles were evaluated using one-way analysis of variance (ANOVA) followed by Bonferroni-adjusted post hoc comparisons for normally distributed variables. Effect sizes were quantified using eta-squared (η^2^). For side effect frequency and side effect severity, which were not normally distributed, the Kruskal–Wallis test was used.

Sensitivity analyses were repeated after excluding participants with very low habitual caffeine intake (<60 mg/day). Additional sensitivity models incorporated body mass as a covariate, with habitual caffeine intake and DAR entered sequentially to examine whether the incremental explanatory value of DAR was robust to adjustment for body mass. These analyses were considered robustness assessments rather than independent validation of the DAR framework.

To assess potential sequence-related and residual effects in the 2 × 2 crossover design, linear mixed-effects models were fitted separately for each outcome. The Sequence effect was examined as an indicator of potential sequence-related differences; however, because sequence is intrinsically related to treatment order in a two-period crossover design, a non-significant Sequence effect was not considered definitive evidence that carry-over was absent. As an additional diagnostic assessment, outcomes measured during Period 2 were compared between the two treatment sequences. Independent-samples t tests were used for continuous performance outcomes, with Welch’s correction applied when the homogeneity-of-variance assumption was violated. For side effect outcomes that were non-normally distributed or ordinal, Mann–Whitney U tests were used. These Period-2 comparisons were interpreted as evidence compatible with potential residual/carry-over effects when significant sequence-related differences were observed.

## 3. Results

### 3.1. Participant Characteristics

Of the 75 participants initially enrolled, 72 completed all study procedures and were included in the final analyses, with a mean age of 25.69 ± 4.83 years and a body mass of 86.92 ± 20.86 kg. Three participants were excluded because they did not complete the study (one due to an unforeseen travel commitment and two due to illness unrelated to the intervention). Wrestlers represented both freestyle (44.4%) and Greco-Roman (55.6%) wrestling disciplines and included amateur (40.3%), semi-professional (38.9%), and professional (20.8%) athletes. Mean training history and weekly training volume were 10.25 ± 2.70 years and 6.63 ± 1.21 h per week, respectively (Table 1). Moreover, the effects of caffeine on performance and side effects are presented in the Supplementary File.

### 3.2. Comparison of Habitual Caffeine Intake-Based Indices

Across the study sample, the mean DAR was 2.54 ± 1.66, indicating that the administered caffeine dose was, on average, approximately 2.5 times higher than participants’ habitual daily caffeine intake. The mean DDI was 173.91 ± 110.41 mg, reflecting substantial inter-individual variation in the absolute difference between the experimental dose and habitual caffeine consumption. The DNI averaged 0.47 ± 0.26, suggesting that, on average, participants’ habitual caffeine intake corresponded to approximately half of the administered dose, although considerable variability was observed among individuals (Table 1). Of the 72 participants, two reported a habitual caffeine intake below 30 mg/day and were assigned the floor value for DAR calculation.

### 3.3. Associations Between Personalized Indices and Ergogenic Response

Correlation analyses were conducted to examine the associations between the personalized caffeine indices and the prespecified primary outcomes (ΔTn, ΔVJ, and CUI), as well as secondary performance and adverse-effect outcomes. Given the exploratory nature of this secondary analysis, these associations were interpreted as measures of explanatory association rather than predictive validity.

**Figure 2:**
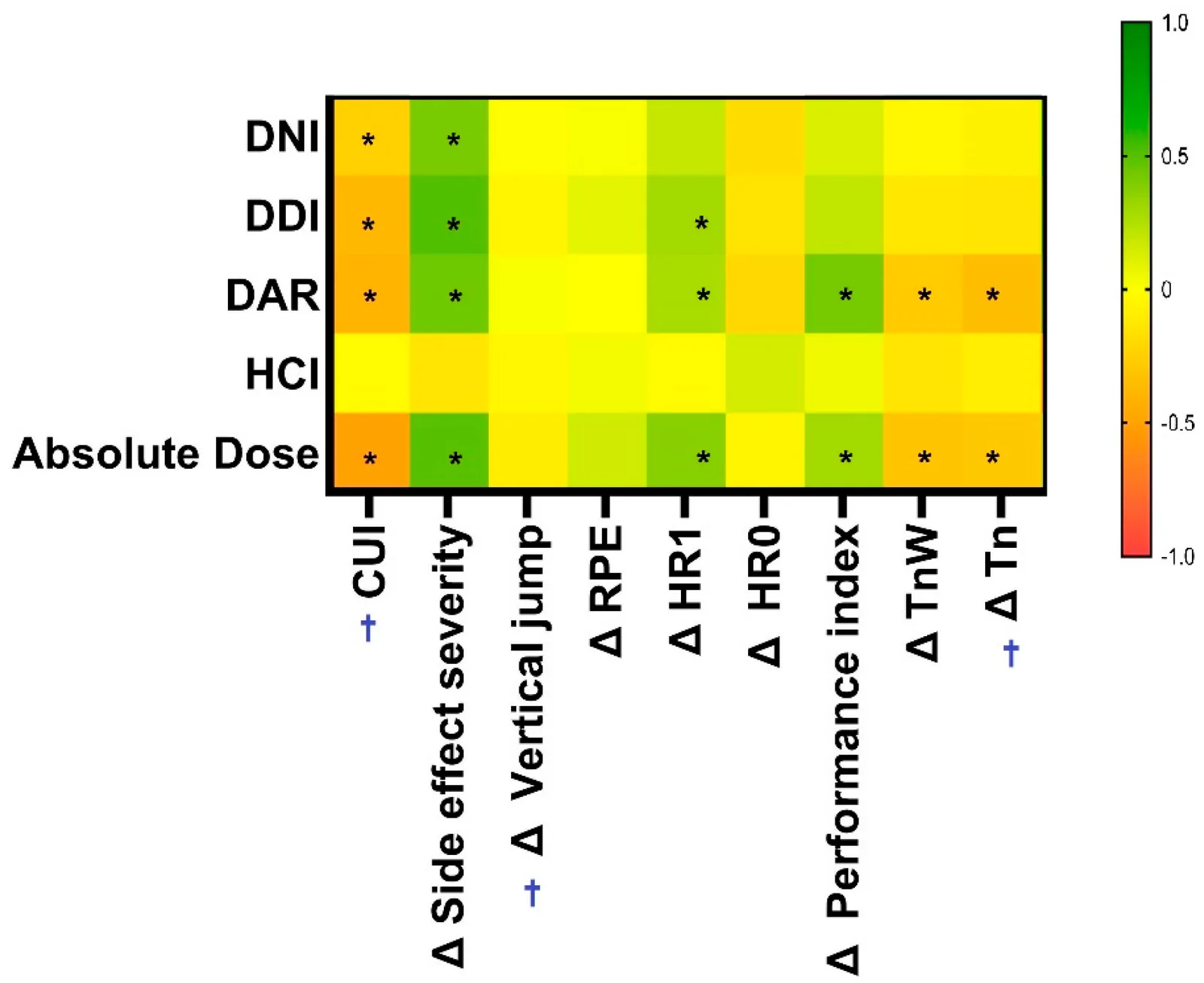
Correlation Heatmap of Traditional Caffeine Variables, Personalized Caffeine Indices, and Ergogenic Outcomes. The heatmap presents correlation coefficients between traditional caffeine exposure variables, personalized caffeine indices, and the study outcomes. Prespecified primary outcomes were ΔTn, ΔVJ, and CUI; other performance and adverse-effect measures were considered secondary outcomes. Color intensity represents the strength and direction of the correlation coefficients, ranging from −1 (red, strong negative correlation) to +1 (green, strong positive correlation), with yellow indicating weak or no correlation. DAR: Dose-to-Habitual Intake Ratio. DDI: Dose Difference Index. DNI: Dose Novelty Index. HCI: habitual caffeine intake. ΔTn: Change in number of throws. ΔTnW: weight-adjusted number of throws. ΔRPE: changes in rating of perceived exertion; ΔHR1: changes in heart rate immediately after the test; ΔHR0: changes in heart rate after 1 min of passive recovery. CUI: Caffeine Utility Index. † Prespecified primary outcomes: ΔTn, ΔVJ, and CUI. * Significant correlation (*p* < 0.05).

DAR were associated with improvements in Tn (r = −0.346, *p* = 0.003) and wrestling performance index (r = 0.423, *p* = 0.001). In contrast, neither the DDI nor the DNI showed significant correlations with improvements in wrestling performance indexes (all *p* > 0.05) (Figure 2).

The composite CUI was also considered a primary exploratory outcome. CUI was negatively associated with DAR (r = −0.234, *p* = 0.047), DDI (r = −0.365, *p* = 0.002), and DNI (r = −0.309, *p* = 0.009). Because higher CUI values represent a more favorable balance between ergogenic benefit and adverse effects, these inverse associations indicate that greater values of the personalized exposure indices were associated with lower CUI values in this sample.

Side effect severity was positively associated with DAR (Spearman’s ρ = 0.351, *p* = 0.002), DDI (ρ = 0.419, *p* < 0.001), and DNI (ρ = 0.412, *p* < 0.001). Similarly, absolute caffeine dose was positively associated with side effect severity (ρ = 0.370, *p* = 0.001). These findings indicate that greater values of the caffeine exposure indices were associated with greater reported adverse-effect burden; however, because adverse effects were assessed retrospectively after the exercise test, these associations cannot establish whether reported symptoms were attributable specifically to caffeine, exercise, or their combination (Figure 2).

### 3.4. Incremental Explanatory Value of the Personalized Caffeine Indices

Hierarchical regression analyses were conducted to examine whether the DAR, DDI and DNI provided incremental explanatory value beyond participant characteristics, habitual caffeine intake, and absolute caffeine dose.

#### 3.4.1. DAR

For ΔTn, the baseline model explained 19.2% of the variance (R^2^ = 0.192, adjusted R^2^ = 0.130; *p* = 0.013). Adding habitual caffeine intake and absolute caffeine dose increased the explained variance by 1.8% (ΔR^2^ = 0.018, *p* = 0.490; total R^2^ = 0.210). In contrast, addition of DAR produced a substantial and statistically significant increase in explained variance (ΔR^2^ = 0.249, *p* < 0.001), resulting in a total R^2^ of 0.458 (adjusted R^2^ = 0.389). In the final model, DAR showed a negative adjusted association with ΔTn (B = −1.512, β = −1.098, *p* < 0.001). However, because DAR and habitual caffeine intake were substantially collinear (VIF = 4.85), the coefficient should be interpreted as an adjusted association within the multivariable model rather than as an independent causal effect (Table 2).

For ΔVJ, the baseline model explained only 6.2% of the variance and was not statistically significant (R^2^ = 0.062, adjusted R^2^ = −0.009; *p* = 0.503). Adding habitual caffeine intake and absolute caffeine dose increased R^2^ by only 1.6% (ΔR^2^ = 0.016, *p* = 0.573; total R^2^ = 0.078). The subsequent addition of DAR produced a further 0.4% increase in explained variance, which was also not statistically significant (ΔR^2^ = 0.004, F-change = 0.299, *p* = 0.586), resulting in a final R^2^ of 0.083. DAR was not significantly associated with ΔVJ in the final model (B = 0.136, β = 0.145, *p* = 0.586).

For CUI, the participant-characteristics model accounted for 8.2% of the variance and was not statistically significant (R^2^ = 0.082, adjusted R^2^ = 0.012; *p* = 0.329). The addition of habitual caffeine intake and absolute caffeine dose significantly increased explained variance by 15.9% (ΔR^2^ = 0.159, *p* = 0.002, total R^2^ = 0.241). Adding DAR produced a further 16.0% increase in explained variance (ΔR^2^ = 0.160, *p* < 0.001), with the final model explaining 40.0% of the variance (adjusted R^2^ = 0.324; *p* < 0.001). DAR was negatively associated with CUI after adjustment for the other variables (B = −1.017, β = −0.879, 95% CI: −1.513 to −0.521, *p* < 0.001), indicating that higher DAR values were associated with lower CUI values within the adjusted model. Given the substantial overlap between DAR and HCI (VIF = 4.847 and 4.746, respectively), this association should be interpreted cautiously and primarily as incremental explanatory value rather than evidence of an independent effect (Table 2).

#### 3.4.2. DDI

For ΔTn, traditional characteristics explained 18.6% of the variance (R^2^ = 0.186, *p* = 0.020), whereas habitual caffeine intake and absolute caffeine dose did not provide a significant increment (ΔR^2^ = 0.018, *p* = 0.491). Adding DDI increased explained variance by 6.1% (ΔR^2^ = 0.061, *p* = 0.027), with the final model explaining 26.5% of the variance (adjusted R^2^ = 0.169, *p* = 0.011) (Table 2).

For ΔVJ, neither the traditional variables (R^2^ = 0.054, *p* = 0.599) nor the addition of habitual caffeine intake and absolute caffeine dose (ΔR^2^ = 0.016, *p* = 0.596) significantly improved model fit. DDI provided no additional explanatory value (ΔR^2^ = 0.002, *p* = 0.716), and the final model was not significant (R^2^ = 0.072, adjusted R^2^ = −0.050, *p* = 0.780).

For CUI, the traditional variables explained 7.5% of the variance (R^2^ = 0.075, *p* = 0.403). Adding habitual caffeine intake and absolute caffeine dose increased explained variance by 16.2% (ΔR^2^ = 0.162, *p* = 0.003), whereas the additional contribution of DDI was not statistically significant (ΔR^2^ = 0.037, *p* = 0.084). The final model explained 27.3% of the variance (adjusted R^2^ = 0.178, *p* = 0.009) (Table 2).

#### 3.4.3. DNI

The incremental contribution of DNI was examined for ΔTn, ΔVJ, and CUI after adjustment for age, training history, competition level, wrestling style, habitual caffeine intake, and absolute caffeine dose. Adding DNI to the regression models did not significantly improve the explained variance for ΔTn (ΔR^2^ = 0.010, *p* = 0.393), ΔVJ (ΔR^2^ = 0.001, *p* = 0.823), or CUI (ΔR^2^ = 0.006, *p* = 0.503). In the final models, DNI was not independently associated with ΔTn (β = −0.382, *p* = 0.393), ΔVJ (β = −0.109, *p* = 0.823), or CUI (β = −0.294, *p* = 0.503). Thus, within the adjusted models, DNI did not explain additional variability in these performance-related outcomes (Table 2).

As a supplementary analysis, hierarchical regression models examined the incremental explanatory value of personalized caffeine indices beyond traditional participant characteristics. These analyses showed that DAR provided significant incremental explanatory value for ΔTn, CUI, and change in wrestling performance index, whereas no significant incremental contribution was observed for ΔVJ (Table S2); full results for DAR, DDI, DNI, habitual caffeine intake, and absolute caffeine dose are provided in the Supplementary File.

#### 3.4.4. Exploratory Identification and Internal Assessment of DAR Response Patterns

Because DAR showed the largest observed incremental explanatory value for outcomes, it was selected for further exploratory analyses. In contrast, neither DDI nor DNI significantly improved the prediction of outcomes in the hierarchical regression models (Table S2). To examine whether the relationship between DAR and ergogenic response was linear or curvilinear, quadratic regression analyses were performed using centered DAR values and their squared term.

**Figure 3:**
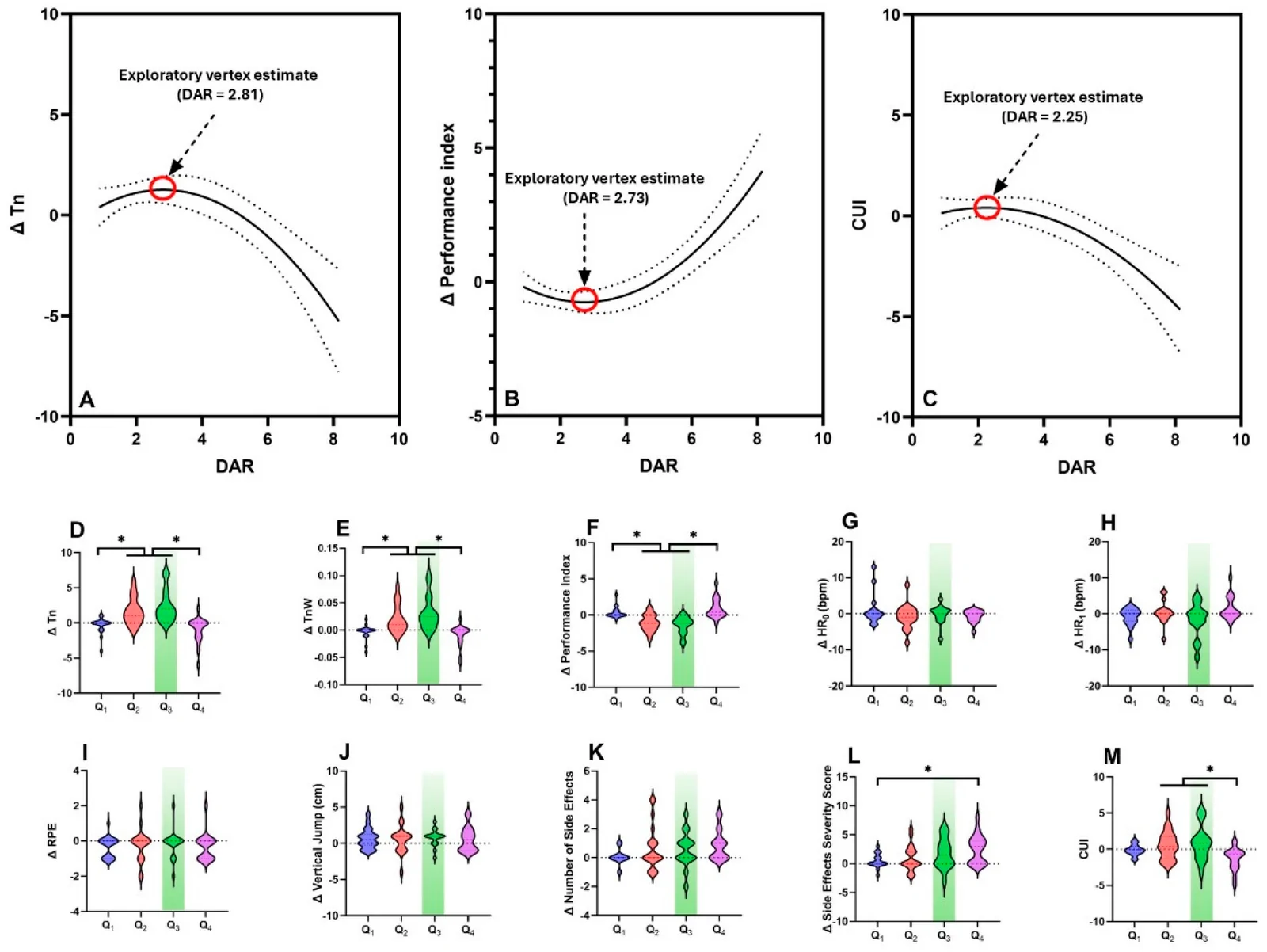
Exploratory Identification of DAR Response Patterns for Ergogenic Performance and Caffeine Utility. The upper panels (**A**–**C**) illustrate the quadratic relationships between DAR and ΔTn, the wrestling performance index, and the CUI. Solid lines represent the fitted quadratic regression models, and dotted lines indicate the 95% confidence intervals. Red circles denote the exploratory vertex estimates derived from each quadratic model. These vertices should be interpreted as sample-specific exploratory estimates rather than validated optimal values. Lower values of the wrestling performance index indicate more favorable outcomes. The lower panels (**D**–**M**) present the distribution of performance, physiological, and perceptual outcomes across DAR quartiles (Q1–Q4). Participants were categorized into quartiles as follows: Q1 (≤1.38), Q2 (1.39–2.00), Q3 (2.01–3.10), and Q4 (≥3.11). Each violin plot combines a kernel density estimate with an embedded box plot (median and interquartile range) and overlaid individual data points, allowing simultaneous visualization of the distribution, central tendency, dispersion, and individual observations within each quartile. Violin width reflects the relative density of observations at each value of the outcome. The green shaded area in the lower panels indicates the Q3 quartile, which corresponds to the exploratory vertex estimate range (or optimal range) derived from the quadratic models. HR_0_: Heart rate immediately after the test. HR_1_: Heart rate 1 min after the test. RPE: Rate of perceived exertion. CUI: Caffeine Utility Index. DAR: Dose-to-Habitual Intake Ratio. ΔTn: Change in number of throws. ΔTnW: Weight-adjusted number of throws. * Significant pairwise differences identified by Bonferroni post hoc comparisons (*p* < 0.05).

For the ΔTn, the quadratic model was significant (R^2^ = 0.239, F = 10.86, *p* < 0.001). The linear DAR term was not significant (β = 0.089, *p* = 0.599), whereas the quadratic term was significant (β = −0.556, *p* = 0.002), supporting an inverted U-shaped association between DAR and throwing performance. The estimated vertex of the quadratic function was DAR = 2.81 (Figure 3), indicating that the modeled response was maximal at approximately 2.8 times the athlete’s habitual caffeine intake.

A similar curvilinear association was observed for the wrestling performance index (R^2^ = 0.333, F = 17.19, *p* < 0.001). The quadratic term was significant (β = 0.631, *p* < 0.001), whereas the linear component was not (β = −0.071, *p* = 0.653). The estimated turning point of the quadratic model occurred at a DAR of approximately 2.73 (Figure 3). The CUI also demonstrated a significant quadratic association with DAR (R^2^ = 0.234, F = 10.52, *p* < 0.001). The quadratic component was significant (β = −0.423, *p* = 0.015), whereas the linear term was not (β = −0.074, *p* = 0.665), with the estimated vertex occurring at approximately DAR = 2.25 (Figure 3).

To provide an additional, distribution-based assessment of these modeled patterns, participants were categorized into four DAR quartiles (≤1.38, 1.39–2.00, 2.01–3.10, and >3.10), and performance outcomes were compared across quartiles. Significant between-quartile differences were observed for the change in number of throws (F = 13.76, *p* < 0.001, η^2^ = 0.378) and the wrestling performance index (F = 17.21, *p* < 0.001, η^2^ = 0.432). Bonferroni-adjusted comparisons showed that athletes in the third DAR quartile (2.01–3.10) achieved greater improvements than those in the lowest quartile, whereas performance outcomes were less favorable in the highest quartile (>3.10). This pattern was broadly consistent with the curvilinear associations identified in the quadratic models (Figure 3).

Similarly, CUI differed significantly across DAR quartiles (F = 5.57, *p* = 0.002, η^2^ = 0.197). Athletes in the highest DAR quartile exhibited poorer utility scores than those in the second and third quartiles, indicating a less favorable overall performance–adverse-effect balance at higher relative caffeine exposure. In contrast, no significant between-quartile differences were observed for VJ performance, immediate post-exercise heart rate, recovery heart rate, or perceived exertion (all *p* > 0.05). Side effect frequency and severity did not differ significantly across DAR quartiles (*p* = 0.106 and *p* = 0.052, respectively) (Figure 3).

Collectively, the quadratic models yielded theoretical DAR vertices between 2.25 and 2.81 across the three primary outcomes. Together with the quartile analyses, these findings suggest that intermediate DAR values were associated with more favorable performance and utility profiles. However, this range should be considered hypothesis-generating rather than a practical dosing target, because DAR was not prospectively assigned, only one experimental caffeine dose was administered, and the estimated range was not independently validated.

Finally, bootstrap resampling provided additional evidence for the stability of the estimated quadratic coefficients within the present sample for the change in number of throws (β = −0.228, BCa 95% CI = −0.430 to −0.132, *p* = 0.017), performance index (β = 0.166, BCa 95% CI = 0.073–0.547, *p* = 0.010), and CUI (β = −0.146, BCa 95% CI = −0.248 to −0.077, *p* = 0.003). Bootstrap analyses provided internal evidence that the quadratic coefficients were relatively stable within this sample, but they do not constitute external validation or establish the generalizability of the estimated DAR range to independent populations.

### 3.5. Assessment of Sequence and Potential Carry-Over Effects

Results for the sequence and potential carry-over effects are presented in the Supplementary File (Table S3). Briefly, no significant overall Sequence effects were observed for the performance outcomes (all *p* ≥ 0.353). Consistently, comparisons between sequences during Period 2 showed no significant differences in any of these outcomes (all *p* ≥ 0.180), providing no evidence of a meaningful sequence-related effect on performance. However, the Period-2 comparisons revealed significant differences between the two treatment sequences for both Side Effect (*p* = 0.012) and Severity of Side Effects (*p* = 0.004). These Period-2 sequence differences are compatible with potential residual/carry-over effects on side effect outcomes, although they do not by themselves establish the presence of carry-over.

### 3.6. Sensitivity Analyses

To examine the potential influence of very low habitual caffeine intake on the ratio-based analyses, sensitivity analyses were conducted after excluding participants with habitual caffeine intake < 60 mg/day (excluded participants = 4). The incremental explanatory value of DAR for ΔTn and ΔVJ remained consistent with the primary analyses, whereas its incremental explanatory value for CUI was attenuated but remained statistically significant (ΔR^2^ = 0.055, *p* = 0.028). Detailed results are presented in the Supplementary File (Table S4). These findings suggest that the main DAR-related associations were not uniformly driven by participants with very low habitual caffeine intake.

Adding body mass to the baseline model did not materially alter the overall pattern of findings. After adjustment for body mass and habitual caffeine intake, DAR retained significant incremental explanatory value for ΔTn (ΔR^2^ = 0.246, *p* < 0.001) and CUI (ΔR^2^ = 0.167, *p* < 0.001). In contrast, DAR did not provide significant incremental explanatory value for ΔVJ (ΔR^2^ = 0.004, *p* = 0.621). Detailed results are presented in the Supplementary File (Table S5). The DAR coefficients and ΔR^2^ values for ΔTn and CUI were also very similar to those observed in the primary models, supporting the robustness of the findings to adjustment for body mass.

## 4. Discussion

The main contribution of the present study is the proposal and comparative evaluation of the DAR as a simple way to characterize caffeine exposure beyond the traditional mg·kg^−1^ prescription. Conventional body-mass dosing standardizes the amount administered relative to body size, but it does not indicate how novel that dose is for a particular athlete. The same 4 mg·kg^−1^ dose can represent a modest increment above usual intake for a habitual consumer and a markedly greater exposure for an athlete with low habitual intake. By expressing the administered dose as a multiple of habitual intake, DAR integrates two quantities that are commonly considered separately. In the current dataset, DAR showed the largest observed explanatory value for wrestling-specific performance among the candidate indices, whereas DDI showed the largest observed ΔR^2^ for side effect severity. This pattern suggests that relative and absolute departures from habitual exposure may be relevant to different aspects of the caffeine response [5,7,14]. From a practitioner’s perspective, this distinction is intuitive: mg·kg^−1^ standardizes dose for body size, but it does not standardize how familiar or novel that dose is to the athlete.

### 4.1. Ability of the Indices to Characterize the Ergogenic Response to Caffeine

Among the candidate indices, DAR showed the largest observed explanatory value for the wrestling-specific outcomes. Importantly, in hierarchical models that included both habitual caffeine intake and absolute caffeine dose (Model 2), DAR still provided significant incremental explanatory value for ΔTn (ΔR^2^ = 0.249, *p* < 0.001), the performance index (ΔR^2^ = 0.321, *p* < 0.001), CUI (ΔR^2^ = 0.160, *p* < 0.001), and side effect severity (ΔR^2^ = 0.072, *p* = 0.008). DAR captured additional explanatory information beyond the linear contributions of its constituent variables in this dataset, although this does not exclude effects arising from its ratio-based mathematical structure. A 300-mg dose, for example, represents a sixfold exposure relative to a habitual intake of 50 mg·day^−1^ but only a 1.2-fold exposure for an athlete habitually consuming 250 mg·day^−1^ [5,11,12,22,23,24,25]. This does not mean that habitual exposure determines the response, but it provides a biologically and practically plausible context in which to interpret it.

The performance findings should nevertheless be interpreted as explanatory and hypothesis-generating rather than as evidence that DAR is already a validated predictor. The study was a secondary analysis of a trial in which all athletes received the same relative dose (4 mg·kg^−1^); DAR was not prospectively assigned. In addition, DAR is a ratio and is therefore sensitive to very low habitual intake values. The apparent association between DAR and performance may partly reflect the ratio-variable structure rather than a novel biological interaction, because DAR combines administered dose (numerator, determined by body mass) and habitual intake (denominator, estimated from the FFQ). We addressed this by comparing DAR with models containing both constituent variables simultaneously, although the moderate collinearity between DAR and habitual intake (VIF = 4.85) should be considered. The absence of a meaningful association with vertical-jump response further suggests outcome specificity: a habitual-exposure metric may be more relevant to repeated, fatigue-sensitive wrestling performance than to a brief isolated explosive task [18,28,29,30]. A related consideration is that, because the administered caffeine dose was calculated as 4 mg·kg^−1^ body mass, absolute caffeine dose is mathematically related to body mass. Body mass was therefore not included as a separate covariate in the primary regression models to avoid collinearity with absolute caffeine dose. However, sensitivity analyses with body mass included in the baseline model yielded materially similar results, supporting the robustness of the DAR-related findings to adjustment for body size. This is particularly relevant in wrestlers, in whom body mass and weight category may influence both performance and caffeine dosing.

Exploratory quadratic analyses suggested that the relationship between DAR and wrestling-specific outcomes may not be strictly linear, with theoretical vertices in the approximate range of 2–3. Quartile analyses were directionally consistent with this pattern, with the third quartile showing the most favorable wrestling-specific responses. However, these analyses were conducted in the same sample used to derive the indices, so the resulting range should be viewed as a sample-specific hypothesis rather than a prescription threshold. Prospective trials in which athletes are assigned to predefined DAR targets are required to determine whether deliberately targeting an intermediate ratio improves performance.

### 4.2. Ability of the Indices to Characterize Side Effects After Caffeine Supplementation

The indices showed a different pattern for tolerability. DDI showed a numerically larger observed ΔR^2^ for side effect severity (ΔR^2^ = 0.231) than absolute caffeine dose (ΔR^2^ = 0.211), suggesting that the absolute amount of caffeine administered above the athlete’s usual intake may be relevant to acute symptom burden. However, the difference between DDI and absolute caffeine dose was small (2.0%) and was not formally tested, so this comparison should be interpreted descriptively rather than as evidence that DDI clearly outperforms absolute dose. Whereas DAR expresses exposure as a relative multiple, DDI quantifies the additional caffeine burden in milligrams. Two athletes can therefore have similar DAR values while receiving very different absolute doses. This distinction may help explain why DDI was more strongly related to side effect severity than to wrestling performance [14,39,40,41,42]. In competition, this trade-off matters because an ergogenic gain can lose practical value when it is accompanied by symptoms that disturb comfort, concentration, or subsequent sleep. However, it should be emphasized that side effects were assessed retrospectively after exhaustive exercise, and the reported symptoms may reflect a combination of caffeine-related and exercise-related effects.

DAR and DNI were also associated with side effect severity, indicating that tolerability may reflect both absolute and relative departures from habitual exposure. Importantly, DNI is algebraically related to DAR and should not be viewed as a fully independent source of information. The side effect findings therefore support the broader principle that habitual exposure should be considered alongside absolute dose, but they do not establish three separate biological pathways or three independent prescription indices [5,22,23,24,25,39,40,41].

### 4.3. Ability of the Indices to Characterize the Caffeine Utility Index

The CUI was developed to integrate performance benefit and adverse-effect severity within a single exploratory measure. Under the stated equation, higher CUI values indicate more favorable utility because standardized performance changes are added and standardized side effect severity is subtracted. DAR provided significant additional explanatory value for CUI, whereas DDI and DNI did not significantly improve the model beyond habitual caffeine intake and absolute caffeine dose. DAR has the advantage of being immediately interpretable as a multiple of habitual intake; DDI, in contrast, retains the clinically intuitive unit of milligrams and may be especially relevant to tolerability.

The selection of ΔTn and ΔVJ as performance components was based on the a priori designation of these variables as primary outcomes of the study. The wrestling Performance Index was not included to avoid redundancy, as it is derived from the same performance test as ΔTn. The CUI should be treated as an exploratory, internally constructed outcome rather than a validated decision instrument. Its components were standardized within the present sample and equally weighted, although athletes may value a change in performance and a change in adverse symptoms differently. Future work should therefore examine athlete-centered weighting, reliability, and external validity before using the CUI to guide decisions.

### 4.4. Practical Interpretation and the Proposed DAR Framework

**Figure 4:**
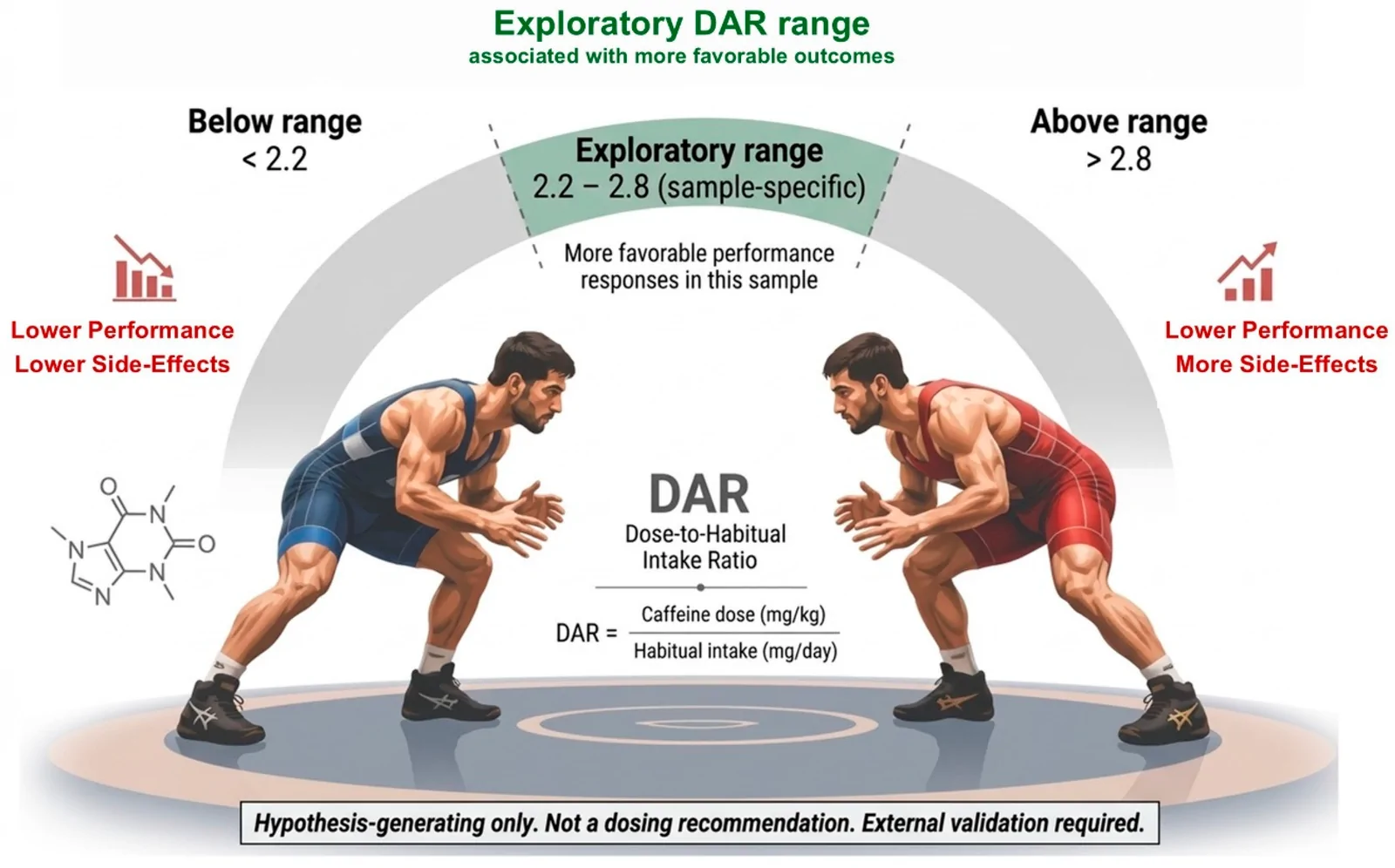
Conceptual summary of the exploratory DAR range associated with more favorable outcomes in this sample and its association with performance and side effect outcomes. DAR values between approximately 2.2 and 2.8 were associated with favorable performance responses, whereas values below 2.2 (low DAR) corresponded to lower performance and fewer side effects, and values above 2.8 (high DAR) were associated with lower performance and more pronounced side effects. DAR, Dose-to-Habitual Intake Ratio: administered caffeine dose (mg) divided by habitual daily caffeine intake (mg/day).

Taken together, the findings support DAR as the principal candidate for future evaluation because it directly answers the practical question posed by this study: how large is the pre-exercise caffeine exposure relative to what the athlete normally consumes? The exploratory range of approximately 2–3 can be presented as a testable hypothesis and is summarized conceptually in Figure 4, but it should not be interpreted as a current dosing recommendation. Because all participants received 4 mg·kg^−1^, this study cannot establish that changing the dose to force an athlete into a target DAR will reproduce the observed pattern. In particular, athletes with high habitual intake could require very high absolute doses to reach a DAR of 2–3, which may be inappropriate because adverse effects increase with dose and higher doses do not necessarily provide greater ergogenic benefit [14,39].

A more appropriate practical framework is therefore to use DAR, if at all, as a complement to established dose and safety considerations rather than as a replacement for them. Athletes should first identify the lowest effective acute caffeine dose during training while considering habitual intake, prior response, time of day, sleep, anxiety, gastrointestinal symptoms, and competition demands. The present results generate the hypothesis that the relationship between acute and habitual exposure deserves consideration; they do not justify increasing habitual intake or exceeding established caffeine-supplementation ranges to achieve a numerical DAR target [11,14,42]. Put simply, DAR is best considered an additional lens on caffeine exposure, not a numerical target that athletes should chase.

### 4.5. Strengths, Limitations, and Future Research

The randomized, double-blind, placebo-controlled crossover design, the use of trained wrestlers, standardized supplementation and testing, and simultaneous assessment of performance and adverse effects are important strengths. At the same time, the work is exploratory.

Several limitations should be acknowledged: (1) no formal adjustment for multiplicity was applied across the large number of statistical comparisons; (2) the apparent association between DAR and performance may partly reflect the ratio-variable structure (denominator and/or numerator) rather than a novel biological interaction; (3) the floor value of 30 mg/day may attenuate the true variability in DAR among participants with very low habitual intake; (4) the exploratory DAR range was derived in the same dataset from which the index was selected, and these procedures do not constitute independent validation or cross-validation; (5) the 24-h caffeine abstinence period may have induced withdrawal symptoms in habitual consumers, which were not formally assessed; (6) measurement error in the adapted FFQ propagates directly into DAR and DDI; and (7) body mass was not included as a covariate in the primary regression models because absolute caffeine dose was calculated as 4 mg·kg^−1^ body mass, although sensitivity analyses with body mass included in the baseline model yielded materially similar results.

Although formal tests for sequence and period effects were performed (Section 3.6, Supplementary Table S2), the absence of statistically significant sequence effects for performance outcomes should not be interpreted as definitive evidence that carry-over was absent. With the current sample size, the study may have been underpowered to detect small residual effects, and carry-over cannot be entirely excluded. In addition, significant Period-2 differences were observed for side effect outcomes, suggesting that residual effects may have influenced the side effect findings. These diagnostic analyses were exploratory, and no adjustment for multiple comparisons was applied.

Side effects were assessed using a retrospective self-report questionnaire administered after exhaustive exercise, so the questionnaire did not capture the time of symptom onset or duration, and symptoms attributable to caffeine cannot be distinguished from those induced by exhaustive exercise. Larger independent cohorts should test whether DAR adds explanatory information beyond administered dose and habitual intake entered separately, whether the apparent intermediate range is reproducible, and whether any advantage generalizes to women and to other endurance, team, strength, and combat sports [8,9,11,16,22,23,24,25,39,40,41,42,43,44].

## 5. Conclusions

DAR emerged as the candidate index with the largest observed explanatory value for characterizing wrestling-specific ergogenic response within the analytical framework used in this study, while DDI showed the largest observed ΔR^2^ for side effect severity. Importantly, DAR does not replace the traditional body-mass-based dose (mg·kg^−1^); rather, the two approaches are complementary. Body-mass-based dosing remains the established method for determining the absolute caffeine dose, and DAR should be viewed only as a supplementary reference dimension that provides additional information about how novel the acute exposure is relative to the athlete’s habitual caffeine intake. DAR cannot be used alone to determine a caffeine supplementation plan without first establishing a safe upper limit for the acute dose. Exploratory analyses suggested that intermediate DAR values of approximately 2–3 were associated with a more favorable performance–tolerability profile in this sample (Figure 4); however, this range is hypothesis-generating and sample-specific, not a dosing recommendation. Prospective trials and independent external validation are required before DAR can be used to individualize caffeine prescription, and any practical application should prioritize the lowest effective acute dose within established safety limits rather than targeting a specific DAR value.

### Potential Future Application of the DAR Framework

DAR may complement conventional body-mass-based caffeine prescription by adding information about habitual exposure, but it cannot replace it and should be applied only within established safety and efficacy principles. The traditional mg·kg^−1^ dose remains the primary method for determining the absolute caffeine dose, and DAR provides only a supplementary reference dimension. The current study used a fixed dose of 4 mg·kg^−1^ and did not prospectively dose athletes to a target DAR; therefore, the examples below are conceptual rather than prescriptive. Within commonly studied pre-exercise caffeine doses, an athlete with low habitual intake will reach a higher DAR at a lower absolute dose than an athlete with high habitual intake. This feature may help practitioners recognize when a standard mg·kg^−1^ dose represents an unusually large departure from usual exposure and therefore warrants greater attention to tolerability. Conversely, a high habitual consumer may have a low DAR despite receiving a substantial absolute dose, but this should not be interpreted as a reason to automatically increase the dose. The practical concept is summarized in Figure 4.

The DAR framework should also not be interpreted as a recommendation to manipulate habitual caffeine intake simply to achieve a target ratio. Maintaining habitual intake at a level that does not promote unnecessary dependence or tolerance may be reasonable, but abrupt pre-competition withdrawal can produce headache, fatigue, and reduced alertness and has not consistently been shown to enhance the ergogenic effect of an acute caffeine dose [26]. Accordingly, future DAR-guided strategies should prioritize the lowest effective acute dose, individual tolerability, and prospective testing rather than escalating caffeine intake to reach a numerical ratio.

## Figures and Tables

**Table 1:** Participant Characteristics and Caffeine-Related Variables (*n* = 72).

	Mean ± SD	Median (IQR)	Range
Style, *n* (%)			
Freestyle	32 (44.4)	-	-
Greco-Roman style	40 (55.6)	-	-
Level, *n* (%)			
Amateur	29 (40.3)	-	-
Semi-professional	28 (38.9)	-	-
Professional	15 (20.8)	-	-
Age (yrs)	25.69 ± 4.83	25.10 (7.26)	19.25
Training history (yrs)	10.25 ± 2.7	10.15 (3.82)	13.50
Training volume (hour/week)	6.63 ± 1.21	6.75 (1.50)	6
Body mass (kg)	86.92 ± 20.86	86.00 (30.25)	82.40
Absolute caffeine dose (mg)	351.13 ± 81.95	344.00 (120.00)	420
Habitual caffeine intake (mg)	180.97 ± 94.53	162.70 (106.20)	328
DAR	2.54 ± 1.65	2.00 (1.87)	7.30
DDI (mg)	173.91 ± 110.41	155.50 (143.17)	472.90
DNI	0.47 ± 0.26	0.50 (0.40)	1.03

DAR: Dose-to-Habitual Intake Ratio. DDI: Dose Difference Index. DNI: Dose novelty index. IQR: interquartile range. SD: standard deviation.

**Table 2:** Incremental contribution of the personalized caffeine indices to the explanation of caffeine-related outcomes.

Outcome	Model 1 Traditional Variables R^2^ (*p*)	Model 2 HCI + Absolute Dose ΔR^2^ (*p*)	Model 3 DAR ΔR^2^ (*p*)	B_(DAR)_ (95% CI)	β_(DAR)_	Final Model R^2^/adj. R^2^ (*p*)
DAR						
ΔTn	0.192 (0.013)	0.018 (0.490)	0.249 (<0.001 *)	−1.512 (−2.074 to −0.950)	−1.098	0.458/0.389 (<0.001 *)
ΔVJ	0.062 (0.503)	0.016 (0.573)	0.004 (0.586)	0.136 (−0.361 to 0.633)	0.145	0.083/−0.034 (0.682)
CUI	0.082 (0.329)	0.159 (0.002)	0.160 (<0.001 *)	−1.017 (−1.513 to −0.521)	−0.879	0.400/0.324 (<0.001 *)
ΔPerformance index	0.184 (0.017)	0.020 (0.458)	0.321 (<0.001 *)	1.106 (0.767 to 1.445)	1.248	0.525/0.465 (<0.001 *)
ΔSide effect severity	0.093 (0.255)	0.230 (0.000 *)	0.072 (0.008 *)	0.809 (0.218 to 1.400)	0.590	0.395/0.318 (<0.001 *)
DDI						
ΔTn	0.186 (0.020)	0.018 (0.491)	0.061 (0.027 *)	−0.025 (−0.047 to −0.003)	−1.184	0.265/0.169 (0.011 *)
ΔVJ	0.054 (0.599)	0.016 (0.596)	0.002 (0.716)	−0.003 (−0.020 to 0.014)	−0.216	0.072/−0.050 (0.780)
CUI	0.075 (0.403)	0.162 (0.003 *)	0.037 (0.084)	−0.016 (−0.035 to 0.002)	−0.914	0.273/0.178 (0.009 *)
ΔPerformance index	0.173 (0.029)	0.019 (0.494)	0.067 (0.022 *)	0.017 (0.003 to 0.031)	1.239	0.259/0.162 (0.014 *)
ΔSide effect severity	0.093 (0.268)	0.238 (<0.001 *)	0.006 (0.451)	0.008 (−0.013 to 0.029)	0.377	0.337/0.250 (0.001 *)
DNI						
ΔTn	0.186 (0.020)	0.018 (0.491)	0.010 (0.393)	−3.428 (−11.402 to 4.547)	−0.382	0.214/0.110 (0.053)
ΔVJ	0.054 (0.599)	0.016 (0.596)	0.001 (0.823)	−0.660 (−6.531 to 5.210)	−0.109	0.071/−0.051 (0.789)
CUI	0.075 (0.403)	0.162 (0.003 *)	0.006 (0.503)	−2.212 (−8.775 to 4.350)	−0.294	0.242/0.143 (0.023 *)
ΔPerformance index	0.173 (0.029 *)	0.019 (0.494)	0.004 (0.593)	1.388 (−3.775 to 6.550)	0.242	0.196/0.090 (0.084)
ΔSide effect severity	0.093 (0.268)	0.238 (<0.001 *)	0.007 (0.416)	2.990 (−4.314 to 10.293)	0.334	0.338/0.251 (0.001 *)

Hierarchical multiple regression models were used to examine the incremental contribution of the DAR beyond traditional participant characteristics (age, training history, competitive level, and wrestling style), habitual caffeine intake, and absolute caffeine dose. Values for Models 2 and 3 represent ΔR^2^ and the corresponding *p*-value for the change in explained variance. The final-model columns report the total R^2^, adjusted R^2^, and *p*-value for the overall model. * Significant improvements in model fit. B = unstandardized regression coefficient; β = standardized regression coefficient. CI = confidence interval. CUI = Caffeine Utility Index. DAR = Dose-to-Habitual Intake Ratio. DDI = Dose Difference Index. DNI = Dose novelty index. HCI = habitual caffeine intake. ΔTn: Changes in number of throws. ΔVJ: changes in vertical jump.

## Data Availability

Data will be made available on request and subject to approval by the relevant ethics committee.

## Supplementary Materials

The following supporting information can be downloaded at: https://www.mdpi.com/article/10.3390/nu18213625/s1, Figure S1: Effects of caffeine on wrestling-specific performance and side effects; Table S1: Assessment of Blinding Effectiveness Based on Participants’ Treatment Guesses; Table S2: Comparative incremental contribution of traditional caffeine variables and personalized caffeine indices to caffeine-related outcomes; Table S3: Sequence and Period-2 analyses for assessing potential sequence-related effects in the crossover design; Table S4: Sensitivity analyses examining the incremental explanatory value of DAR after excluding participants with habitual caffeine intake <60 mg/day; Table S5: Sensitivity analyses examining the incremental explanatory value of DAR after accounting for body mass and habitual caffeine intake.

## References

[1] Bezuglov E., Vakhidov T., Morgans R., Malyakin G., Emanov A., Koroleva E., Kapralova E., Talibov O. (2025). Caffeine effects on physical performance and sport-specific skills in elite youth soccer players: A randomised trial using the balanced placebo design. Sports.

[2] Qiu B., Wang Z., Zhang Y., Cui Y., Diao P., Liu K., Del Coso J., Liu C. (2025). Effects of caffeine intake combined with self-selected music during warm-up on anaerobic performance: A randomized, double-blind, crossover study. Nutrients.

[3] Negaresh R., Al-Riyami S.A.A., Paahoo A., Hoseini R., Del Coso J. (2025). Effects of Acute Caffeine Ingestion on Physical Performance and Skill Execution in Volleyball Players: A Systematic Review and Meta-Analysis. Int. J. Exerc. Sci..

[4] Maughan R.J., Burke L.M., Dvorak J., Larson-Meyer D.E., Peeling P., Phillips S.M., Rawson E.S., Walsh N.P., Garthe I., Geyer H. (2018). IOC consensus statement: Dietary supplements and the high-performance athlete. Int. J. Sport Nutr. Exerc. Metab..

[5] Carvalho A., Marticorena F.M., Grecco B.H., Barreto G., Saunders B. (2022). Can I Have My Coffee and Drink It? A Systematic Review and Meta-Analysis to Determine Whether Habitual Caffeine Consumption Affects the Ergogenic Effect of Caffeine. Sports Med..

[6] Sutehall S., Pitsiladis Y. (2025). Personalized nutrition for the enhancement of elite athletic performance. Scand. J. Med. Sci. Sports.

[7] Diaz-Lara J., Nieto-Acevedo R., Abian-Vicen J., Del Coso J. (2024). Can caffeine change the game? Effects of acute caffeine intake on specific performance in intermittent sports during competition: A systematic review and meta-analysis. Int. J. Sports Physiol. Perform..

[8] Grgic J., Pickering C., Bishop D.J., Del Coso J., Schoenfeld B.J., Tinsley G.M., Pedisic Z. (2020). ADORA2A C Allele Carriers Exhibit Ergogenic Responses to Caffeine Supplementation. Nutrients.

[9] Guest N.S., Corey P., Vescovi J., El-Sohemy A. (2018). Caffeine, CYP1A2 Genotype, and Endurance Performance in Athletes. Med. Sci. Sports Exerc..

[10] Mielgo-Ayuso J., Marques-Jiménez D., Refoyo I., Del Coso J., León-Guereño P., Calleja-González J. (2019). Effect of Caffeine Supplementation on Sports Performance Based on Differences Between Sexes: A Systematic Review. Nutrients.

[11] Boyett J.C., Giersch G.E.W., Womack C.J., Saunders M.J., Hughey C.A., Daley H.M., Luden N.D. (2016). Time of Day and Training Status Both Impact the Efficacy of Caffeine for Short Duration Cycling Performance. Nutrients.

[12] Khodadadi D., Azimi F., Moghanlou A.E., Gursoy R., Demirli A., Jalali P., Behdari R., Seyedheydari M. (2025). Habitual Caffeine Consumption and Training Status Affect the Ergogenicity of Acute Caffeine Intake on Exercise Performance. Sports Health.

[13] Conway K.J., Orr R., Stannard S.R. (2003). Effect of a divided caffeine dose on endurance cycling performance, postexercise urinary caffeine concentration, and plasma paraxanthine. J. Appl. Physiol..

[14] Guest N.S., VanDusseldorp T.A., Nelson M.T., Grgic J., Schoenfeld B.J., Jenkins N.D.M., Arent S.M., Antonio J., Stout J.R., Trexler E.T. (2021). International society of sports nutrition position stand: Caffeine and exercise performance. J. Int. Soc. Sports Nutr..

[15] Negaresh R., Del Coso J., Mokhtarzade M., Lima-Silva A.E., Baker J.S., Willems M.E.T., Talebvand S., Khodadoost M., Farhani F. (2019). Effects of different dosages of caffeine administration on wrestling performance during a simulated tournament. Eur. J. Sport Sci..

[16] Rohloff G., Souza D.B., Ruiz-Moreno C., DEL Coso J., Polito M.D. (2022). Stimulus Expectancy and Stimulus Response of Caffeine on 4-km Running Performance: A Randomized, Double-blind, Placebo-controlled and Crossover Study. Int. J. Exerc. Sci..

[17] Reyner L.A., Horne J. (2013). Sleep restriction and serving accuracy in performance tennis players, and effects of caffeine. Physiol. Behav..

[18] Del Coso J., Lara B., Ruiz-Moreno C., Salinero J.J. (2019). Challenging the myth of non-response to the ergogenic effects of caffeine ingestion on exercise performance. Nutrients.

[19] Fredholm B.B. (1995). Adenosine, Adenosine Receptors and the Actions of Caffeine. Pharmacol. Toxicol..

[20] Fredholm B.B., Bättig K., Holmén J., Nehlig A., Zvartau E.E. (1999). Actions of Caffeine in the Brain with Special Reference to Factors That Contribute to Its Widespread Use. Pharmacol. Rev..

[21] Svenningsson P., Nomikos G.G., Fredholm B.B. (1999). The stimulatory action and the development of tolerance to caffeine is associated with alterations in gene expression in specific brain regions. J. Neurosci..

[22] Beaumont R., Cordery P., Funnell M., Mears S., James L., Watson P. (2017). Chronic ingestion of a low dose of caffeine induces tolerance to the performance benefits of caffeine. J. Sports Sci..

[23] Gonçalves L.d.S., Painelli V.D.S., Yamaguchi G., Oliveira L.F.D., Saunders B., Da Silva R.P., Maciel E., Artioli G.G., Roschel H., Gualano B. (2017). Dispelling the myth that habitual caffeine consumption influences the performance response to acute caffeine supplementation. J. Appl. Physiol..

[24] Lara B., Ruiz-Moreno C., Salinero J.J., Del Coso J. (2019). Time course of tolerance to the performance benefits of caffeine. PLoS ONE.

[25] Ruiz-Moreno C., Lara B., Gutiérrez-Hellín J., González-García J., Del Coso J. (2020). Time Course and Magnitude of Tolerance to the Ergogenic Effect of Caffeine on the Second Ventilatory Threshold. Life.

[26] Irwin C., Desbrow B., Ellis A., O’KEeffe B., Grant G., Leveritt M. (2011). Caffeine withdrawal and high-intensity endurance cycling performance. J. Sports Sci..

[27] Liu H., Cao Y., Zhang G., Li H. (2026). Beyond Ergogenic Effects: Limited Evidence on Caffeine-Related Problematic Use, Dependence, and Withdrawal in Athletic Populations—A Structured Narrative Review. Nutrients.

[28] Giráldez-Costas V., González-García J., Lara B., Del Coso J., Wilk M., Salinero J.J. (2020). Caffeine Increases Muscle Performance During a Bench Press Training Session. J. Hum. Kinet..

[29] Lara B., Gonzalez-Millán C., Salinero J.J., Abian-Vicen J., Areces F., Barbero-Alvarez J.C., Muñoz V., Portillo L.J., Gonzalez-Rave J.M., Del Coso J. (2014). Caffeine-containing energy drink improves physical performance in female soccer players. Amino Acids.

[30] Muñoz A., López-Samanes Á., Aguilar-Navarro M., Varillas-Delgado D., Rivilla-García J., Moreno-Pérez V., Del Coso J. (2020). Effects of CYP1A2 and ADORA2A Genotypes on the Ergogenic Response to Caffeine in Professional Handball Players. Genes.

[31] Bühler E., Lachenmeier D.W., Schlegel K., Winkler G. (2014). Development of a tool to assess the caffeine intake among teenagers and young adults. Ernahr. Umsch..

[32] Bougrine H., Ammar A., Salem A., Trabelsi K., Jahrami H., Chtourou H., Souissi N. (2024). Effects of various caffeine doses on cognitive abilities in female athletes with low caffeine consumption. Brain Sci..

[33] Karayigit R., Naderi A., Saunders B., Forbes S.C., Del Coso J., Berjisian E., Yildirim U.C., Suzuki K. (2021). Combined but not isolated ingestion of caffeine and taurine improves Wingate Sprint Performance in female team-sport athletes habituated to caffeine. Sports.

[34] Markovic G., Dizdar D., Jukic I., Cardinale M. (2004). Reliability and factorial validity of squat and countermovement jump tests. J. Strength Cond. Res..

[35] de Salles P., Vasconcellos F., de Salles G., Fonseca R., Dantas E. (2012). Validity and reproducibility of the sargent jump test in the assessment of explosive strength in soccer players. J. Hum. Kinet..

[36] Marković M., Kukić F., Dopsaj M., Kasum G., Toskic L., Zaric I. (2021). Validity of a novel specific wrestling fitness test. J. Strength Cond. Res..

[37] Marković M., Toskić L., Kukić F., Zarić I., Dopsaj M. (2022). Sensitivity of field tests for assessment of wrestlers specific fitness. J. Hum. Kinet..

[38] Francino L., Villarroel B., Valdés-Badilla P., Ramirez-Campillo R., Martín E.B.-S., Ojeda-Aravena A., Aedo-Muñoz E., Pardo-Tamayo C., Herrera-Valenzuela T. (2022). Effect of a six week in-season training program on wrestling-specific competitive performance. Int. J. Environ. Res. Public Health.

[39] Wilk M., Krzysztofik M., Filip A., Zajac A., Del Coso J. (2019). The effects of high doses of caffeine on maximal strength and muscular endurance in athletes habituated to caffeine. Nutrients.

[40] Childs E., De Wit H. (2006). Subjective, behavioral, and physiological effects of acute caffeine in light, nondependent caffeine users. Psychopharmacology.

[41] Ding L., Liu J., Yao Y., Guo L., Chen B., Cao Y., Girard O. (2025). Caffeinated chewing gum enhances maximal strength and muscular endurance during bench press and back squat exercises in resistance-trained men. Front. Nutr..

[42] Gardiner C.L., Weakley J., Burke L.M., Fernandez F., Johnston R.D., Leota J., Russell S., Munteanu G., Townshend A., Halson S.L. (2025). Dose and timing effects of caffeine on subsequent sleep: A randomized clinical crossover trial. Sleep.

[43] Grgic J., Pickering C., Bishop D.J., Schoenfeld B.J., Mikulic P., Pedisic Z. (2020). CYP1A2 genotype and acute effects of caffeine on resistance exercise, jumping, and sprinting performance. J. Int. Soc. Sports Nutr..

[44] Puente C., Abián-Vicén J., Del Coso J., Lara B., Salinero J.J. (2018). The CYP1A2-163C>A polymorphism does not alter the effects of caffeine on basketball performance. PLoS ONE.

