# Supplementary material for: Development and Exploratory Evaluation of a Habitual Caffeine Intake-Based Index to Characterize the Ergogenic Response to Caffeine in Wrestlers
Source: Nutrients. 2026 Oct 31;18(21):3625. doi: 10.3390/nu18213625 (PMC13765086; doi:10.3390/nu18213625)
Supplement: Supplementary file 1 [file nutrients-18-03625-s001.zip › nutrients-4557230-supplementary.pdf]

Supplementary File

Blinding Effectiveness

Blinding effectiveness was assessed immediately after each experimental session by asking participants to indicate whether they believed they had received caffeine or placebo. During the caffeine session, 35 of 72 participants (48.6%) correctly identified caffeine, whereas 33 (45.8%) incorrectly identified it as placebo, and 4 (5.6%) were unsure. During the placebo session, 33 of 72 participants (45.8%) correctly identified placebo, whereas 34 (47.2%) incorrectly identified it as caffeine, and 5 (6.9%) were unsure. The Bang Blinding Index was 0.028 for the caffeine session and −0.014 for the placebo session; values close to 0 indicate no substantial deviation from balanced treatment guessing, consistent with successful blinding.

Table S1

Assessment of Blinding Effectiveness Based on Participants' Treatment Guesses

Session	Correct	Incorrect	Don't know	Bang Blinding Index
**Caffeine**	35	33	4	**0.028**
**Placebo**	33	34	5	**−0.014**

Effects of Caffeine on Performance and Side Effect

Compared with placebo, caffeine significantly improved Tn ($P = 0.023$, Cohen's $d = 0.27$) and a weight-adjusted Tn ($P = 0.006$, Cohen's $d = 0.33$) while trend to significantly improve wrestling-specific performance ($P = 0.088$, Cohen's $d = 0.20$). Caffeine additionally significantly increased vertical jump performance ($P = 0.001$, Cohen's $d = 0.41$). No significant differences were observed in heart rate immediately after the test ($P = 0.491$) or after 1 min of recovery ($P = 0.833$). Moreover, Wilcoxon signed-rank test revealed significantly lower RPE ($Z = -2.75$, $P = 0.006$, $r = 0.32$), higher side-effect severity score ($Z = -4.58$, $P < 0.001$, $r = 0.54$) and the number of reported side effects ($Z = -3.64$, $P < 0.001$, $r = 0.43$) following caffeine ingestion vs. placebo condition.

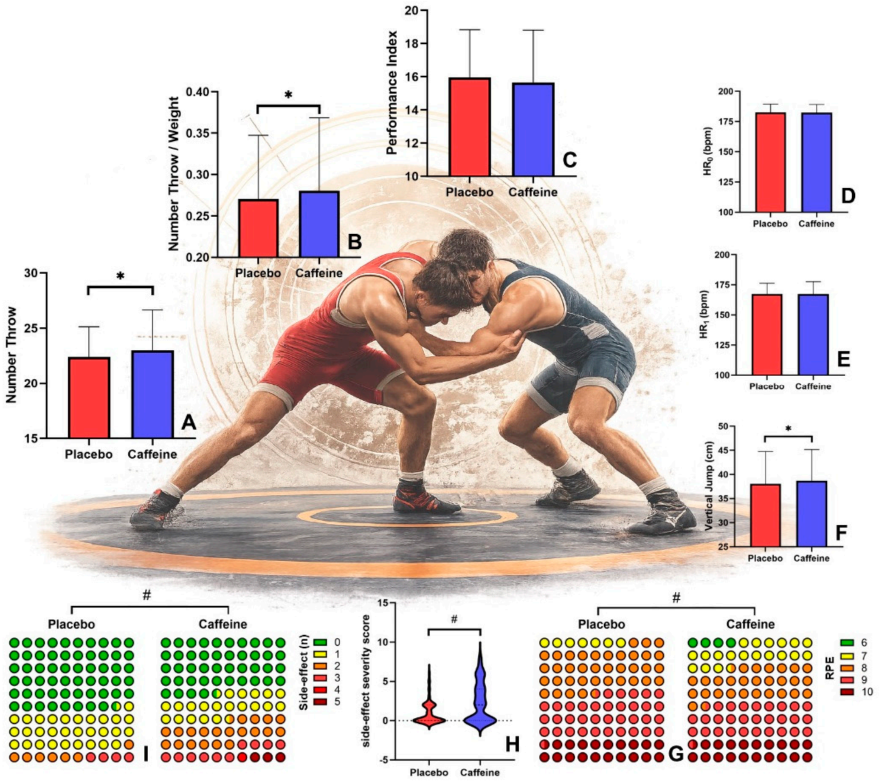

Figure S1. Effects of caffeine on wrestling-specific performance and side effects

Mean ± SD are presented for performance (A-C, F) and physiological variables (D, E). Violin plots display the distribution of side-effect severity scores (H). The frequency distribution of the RPE (G) and number of reported side effects (I) are presented.

HR_0: Heart rate immediately after the test

HR_1: Heart rate 1 min after the test

RPE: Rate of perceived exertion

* Significant difference between caffeine and placebo conditions in paired t-test ($p < 0.05$)

Significant difference between caffeine and placebo conditions in Wilcoxon signed-rank test ($P < 0.01$)

Incremental Explanatory Value Beyond Traditional Variables

Hierarchical regression analyses were performed to determine whether the personalized caffeine indices provided additional explanatory value beyond traditional participant characteristics (age, wrestling style, competitive level, and training history).

For the change in total repetitions (ΔTn), the baseline model explained 16.6% of the variance ($R^2 = 0.166$, $p = 0.015$). Adding absolute caffeine dose did not significantly improve the model ($\Delta R^2 = 0.037$, $p = 0.085$), nor did habitual caffeine intake ($\Delta R^2 = 0.001$, $p = 0.739$), DDI ($\Delta R^2 = 0.013$, $p = 0.324$), or DNI ($\Delta R^2 = 0.003$, $p = 0.636$). In contrast, adding DAR was associated with a significant increase in explained variance of 6.8% ($\Delta R^2 = 0.068$, $p = 0.019$), indicating incremental explanatory value beyond conventional variables (Table 1S).

For the change in vertical jump performance, the baseline model was not significant ($R^2 = 0.018$, $p = 0.873$), and none of the personalized caffeine indices significantly improved the model ($\Delta R^2 = 0.002$–0.015, $p > 0.05$) (Table 2). For the CUI, the baseline model explained 7.6% of the variance ($R^2 = 0.076$, $p = 0.250$). Adding absolute caffeine dose significantly improved the model ($\Delta R^2 = 0.199$, $p < 0.001$), whereas habitual caffeine intake provided no additional explanatory value ($\Delta R^2 < 0.001$, $p = 0.886$). DDI produced the numerically largest observed ΔR^2 ($\Delta R^2 = 0.128$, $p = 0.002$), followed closely by DAR ($\Delta R^2 = 0.122$, $p = 0.002$). DNI also contributed a smaller but statistically significant increase in explained variance ($\Delta R^2 = 0.057$, $p = 0.045$) (Table 1S).

Similarly, for the change in the wrestling performance index, the baseline model explained 16.3% of the variance ($R^2 = 0.163$, $p = 0.017$). The addition of absolute caffeine dose did not significantly improve the model ($\Delta R^2 = 0.041$, $p = 0.071$), nor did habitual caffeine intake ($\Delta R^2 < 0.001$, $p = 0.894$), DDI ($\Delta R^2 = 0.028$, $p = 0.147$), or DNI ($\Delta R^2 = 0.011$, $p = 0.366$). In contrast, DAR was associated with a significant increase in explained variance, accounting for an additional 11.5% of the variance in the change in wrestling performance ($\Delta R^2 = 0.115$, $p = 0.002$) (Table 1S).

For side-effect severity, the baseline model was not significant ($R^2 = 0.048$, $p = 0.499$). Adding absolute caffeine dose significantly improved the model, explaining an additional 21.1% of the variance ($\Delta R^2 = 0.211$, $p < 0.001$). Habitual caffeine intake did not provide incremental explanatory value ($\Delta R^2 = 0.027$, $p = 0.168$). Among the personalized indices, DAR ($\Delta R^2 = 0.168$, $p < 0.001$), DDI ($\Delta R^2 = 0.231$, $p < 0.001$), and DNI ($\Delta R^2 = 0.152$, $p = 0.001$) were all associated with significant increases in explained variance for side-effect severity beyond traditional variables, with DDI showing the largest observed ΔR^2 (Table 1S).

Table S2

Comparative incremental contribution of traditional caffeine variables and personalized caffeine indices to caffeine-related outcomes

Outcome	Baseline R^2	Absolute dose	Habitual intake	DAR	DDI	DNI
**Δ Number of throws**	0.166	0.037 (0.085)	0.001 (0.739)	0.068 (0.019) *	0.013 (0.324)	0.003 (0.636)
**Δ Vertical jumps**	0.018	0.015 (0.313)	0.002 (0.742)	<0.001 (0.919)	0.006 (0.538)	0.002 (0.734)
**CUI**	0.076	0.199 (<0.001) *	<0.001 (0.886)	0.122 (0.002) *	0.128 (0.002) *	0.057 (0.045) *
**Δ Performance index**	0.163	0.041 (0.071)	<0.001 (0.894)	0.115 (0.002) *	0.028 (0.147)	0.011 (0.366)
**Δ Side-effect severity**	0.048	0.211 (<0.001) *	0.027 (0.168)	0.168 (<0.001) *	0.231 (<0.001) *	0.152 (0.001) *

Hierarchical multiple regression analyses evaluating the additional predictive value (ΔR^2) of traditional caffeine-related variables (absolute caffeine dose and habitual caffeine intake) and the proposed personalized caffeine indices (DAR, DDI, and DNI) beyond the baseline model including age, wrestling style, competitive level, and training history. For each predictor, values are presented as ΔR^2 (P-value for ΔR^2 change), where ΔR^2 represents the increase in explained variance after adding the predictor to the baseline model and the value in parentheses corresponds to the significance level of the change in model fit. CUI: Caffeine Utility Index; DAR: Dose-to-Habitual Intake Ratio

DDI: Dose Difference Index

DNI: Dose Novelty Index

* Significant improvements in model fit

Table S3

Sequence and Period-2 analyses for assessing potential sequence-related effects in the crossover design

Outcome	LMM: Sequence F	LMM: p	Period-2 comparison p
**Number of throws**	0.522	.472	.880
**Number of throws/weight**	0.024	.878	.744
**Performance index**	0.358	.552	.867
**Vertical jumps**	0.876	.353	.180
**Side Effect**	0.525	.471	.012
**Severity side effect**	0.384	.538	.004

Table S4

Sensitivity analyses examining the incremental explanatory value of DAR after excluding participants with habitual caffeine intake <60 mg/day

Outcome	Model 1 Traditional variables R^2 (*P*)	Model 2 HCI + Absolute dose ΔR^2 (*P*)	Model 3 DAR ΔR^2 (*P*)	B (DAR) (95% CI)	β (DAR)	Final model R^2 / adj. R^2 (*P*)
**ΔTn**	0.193 (.018)	0.049 (.154)	**0.152 (<.001*)**	−1.740 (−2.643 to −0.837)	−1.009	0.394 / 0.312 (<.001*)
**ΔVJ**	0.056 (.597)	0.019 (.548)	0.041 (.101)	0.663 (−0.134 to 1.461)	0.526	0.116 / −0.003 (.466)
**CUI**	0.077 (.397)	0.214 (<.001*)	**0.055 (.028*)**	−0.732 (−1.382 to −0.082)	−0.570	0.346 / 0.259 (.001*)

Hierarchical multiple regression models were used to examine the incremental contribution of the DAR beyond traditional participant characteristics (age, training history, competitive level, and wrestling style), habitual caffeine intake, and absolute caffeine dos. Values for Models 2 and 3 represent ΔR^2 and the corresponding P-value for the change in explained variance. The final-model columns report the total R^2, adjusted R^2, and P-value for the overall model.

* Significant improvements in model fit

B = unstandardized regression coefficient

β = standardized regression coefficient

CI = confidence interval

CUI = Caffeine Utility Index

DAR = Dose-to-Habitual Intake Ratio

HCI = habitual caffeine intake.

Table S5

Sensitivity analyses examining the incremental explanatory value of DAR after accounting for body mass and habitual caffeine intake

Outcome	Model 1 Traditional variables + body mass R^2 (*P*)	Model 2 HCI ΔR^2 (*P*)	Model 3 DAR ΔR^2 (*P*)	B (DAR) (95% CI)	β (DAR)	Final model R^2 / adj. R^2 (*P*)
**ΔTn**	0.209 (.015)	0.000 (.927)	0.246 (<.001*)	−1.494 (−2.053 to −0.935)	−1.085	0.456 / 0.387 (<.001*)
**ΔVJ**	0.076 (.503)	0.001 (.841)	0.004 (.621)	0.123 (−0.371 to 0.617)	0.131	0.081 / −0.036 (.698)
**CUI**	0.220 (.011)	0.012 (.324)	0.167 (<.001*)	−1.032 (−1.526 to −0.539)	−0.893	0.399 / 0.323 (<.001*)

Hierarchical multiple regression models were used to examine the incremental contribution of the DAR beyond traditional participant characteristics (age, training history, competitive level, and wrestling style), habitual caffeine intake, and absolute caffeine dos. Values for Models 2 and 3 represent ΔR^2 and the corresponding P-value for the change in explained variance. The final-model columns report the total R^2, adjusted R^2, and P-value for the overall model.

* Significant improvements in model fit

B = unstandardized regression coefficient

β = standardized regression coefficient

CI = confidence interval

CUI = Caffeine Utility Index

DAR = Dose-to-Habitual Intake Ratio

HCI = habitual caffeine intake.
